# Optimized Interfacial Layers for High-Adhesion and Damp-Heat-Resistant Cu Meshes with Aperiodic Geometries on PET Substrates

**DOI:** 10.3390/ma19122608

**Published:** 2026-06-17

**Authors:** Xiao Lu, Jia Li, Biyou Bao, Chengli Zhang, Qiang Wang, Guanglong Xu, Xianfa Rao, Hongliang Zhang, Weijie Song

**Affiliations:** 1 School of Materials Science and Engineering, Jiangxi University of Science and Technology, Ganzhou 341000, China; luxiao1@nimte.ac.cn (X.L.);; 2 Ningbo Institute of Materials Technology and Engineering, Chinese Academy of Sciences, Ningbo 315201, China; 3 Ningbo Wakan Electronic Science Technology Co., Ltd., Ningbo 315475, China

**Keywords:** interfacial layer engineering, aperiodic mesh geometry, maskless laser lithography, interfacial adhesion, damp-heat resistance, flexible transparent conductive electrodes

## Abstract

Copper (Cu) thin films and meshes on polyethylene terephthalate (PET) substrates are promising flexible transparent conductive electrodes (TCEs), yet their practical use is limited by insufficient interfacial adhesion and poor oxidative stability on inert polymer substrates. This work addresses these issues via a synergistic strategy of interfacial layer engineering and maskless laser lithography-based aperiodic mesh patterning, systematically comparing ceramic (Al_2_O_3_) and metallic (NiCr) interfacial layers for PET-supported Cu films and fabricating Linear/Sinusoidal aperiodic Cu meshes with tailored performance. Magnetron sputtering shows that Ar plasma-activated NiCr interfacial layers form a gradient-alloyed interface with Cu via interdiffusion, achieving 5B-level adhesion, mitigating bending-induced stress concentration, and enhancing damp-heat resistance (85 °C/85% RH) by suppressing oxidation—outperforming brittle Al_2_O_3_ layers. Patterning the optimized Cu/NiCr/PET structure into micrometer-scale meshes yields a Linear design with superior optoelectronic performance (~10.8 Ω/sq sheet resistance, >87% transmittance at 550 nm) and a Sinusoidal design with enhanced bending robustness via stress delocalization. Microstructural and elemental analyses clarify the NiCr layer’s interfacial toughening and anti-oxidation mechanisms. Practical validation in flexible transparent heaters demonstrates rapid thermal response and >20 h continuous operational stability. This study provides a scalable design strategy for high-performance PET-supported Cu meshes, offering insights for interface and structural optimization of flexible metallic TCEs for next-generation optoelectronics.

## 1. Introduction

Metallic conductive films patterned into micrometer-scale meshes represent a core structural design for flexible transparent conductive electrodes (TCEs), the key components underpinning next-generation optoelectronic devices including flexible displays, wearable sensors, and transparent heaters [1,2,3]. Among earth-abundant metallic materials [4,5,6], copper (Cu) is the most promising candidate for such mesh electrodes, owing to its ultra-high intrinsic electrical conductivity (≈5.8 × 10^7^ S/m) and low cost—advantages that make it ideal for scalable fabrication [7]. However, the practical application of flexible Cu meshes on chemically inert polymer substrates is severely hindered by two critical bottlenecks: insufficient interfacial adhesive strength between Cu and the polymer matrix, and high susceptibility of Cu to oxidative degradation under ambient and harsh service conditions [7,8]. Resolving these issues is essential to improve the electromechanical durability and long-term environmental stability of Cu-based flexible TCEs for practical deployment.

To address the interfacial adhesion and oxidation resistance limitations of Cu mesh electrodes, a variety of modification strategies have been developed, with their action mechanisms centered on chemical anchoring, mechanical embedding, and passivation coating. Chemical anchoring strategies typically introduce refractory metal interfacial layers (e.g., Ti, Cr) [9] or grow oxide interlayers via atomic layer deposition (ALD) [10], where enhanced bonding is achieved through interfacial chemical bond formation. While effective for static interfacial adhesion, the high stiffness of these seed and interlayers easily induces severe stress concentration at the Cu–polymer interface during dynamic small-radius bending, leading to premature crack initiation [11]; additionally, the low deposition rate of ALD makes this approach incompatible with high-throughput manufacturing. Mechanical embedding methods physically submerge conductive Cu meshes into flexible polymer matrices (e.g., photocurable resins) [12], which markedly improves mechanical durability and surface smoothness but adds processing complexity and encapsulates the conductive Cu surface, thus restricting direct electrical contact for out-of-plane optoelectronic applications. Passivation coating strategies employ inorganic oxides (e.g., AZO) [13], 2D materials (e.g., graphene) [14], or conductive polymers (e.g., PEDOT:PSS) [15] as protective overlays, and chemical treatments or polymer capping have also been used to fabricate oxidation-resistant shells for Cu nanowires/particles [16,17]. Though these methods effectively isolate Cu from oxygen and moisture, they often involve complex transfer processes or introduce parasitic optical absorption and haze, which compromise the core optoelectronic performance of transparent electrodes. To fundamentally mitigate interfacial mechanical mismatch and Cu oxidation without sacrificing optical clarity, this work adopts a ductile NiCr alloy interfacial layer strategy, which not only establishes robust interfacial chemical bonding but also acts as a mechanical buffer to dissipate stress during structural deformation.

Beyond interfacial engineering, the macroscopic fabrication method of Cu meshes directly determines the scalability, manufacturing cost, and design flexibility of TCEs. Self-organized cracked templates offer prominent advantages of ultra-low manufacturing cost, mask-free operation, and mature R2R production compatibility [18], and can fabricate semi-embedded Cu meshes with a high Figure of Merit; however, the stochastic nature of crack propagation results in poor design flexibility for customized geometric patterns and localized inconsistencies in patterning resolution, limiting its application in high-precision optoelectronic devices. Conventional photolithography enables fabrication of Cu meshes with exceptional resolution and deterministic geometric uniformity [13] but relies on expensive, rigid physical masks, which increases manufacturing costs and hinders rapid prototyping of novel mesh designs. Maskless laser lithography resolves the design flexibility issue, allowing the construction of complex mesh geometries (e.g., fractal meshes) with excellent performance [19], yet it faces an inherent trade-off between processing throughput and equipment cost. In this study, we prioritize a balanced fabrication approach that achieves high geometric precision, deterministic design flexibility, and precise control over the Cu–polymer interface.

**Figure 1:**
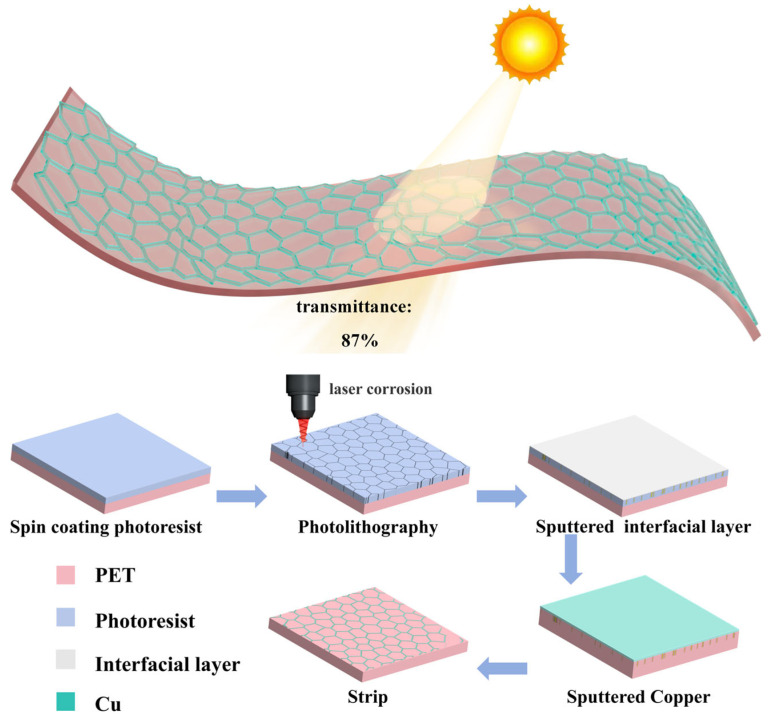
Schematic illustration of the fabrication process and structural design for alloy-engineered Cu mesh electrodes.

This work reports the development of high-performance Cu mesh electrodes via a systematic interfacial engineering strategy, with a rigorous comparative investigation of ceramic (Al_2_O_3_) and metallic (NiCr) interfacial layers for PET-supported Cu films. Activated by in situ Ar plasma, the ductile NiCr interfacial layer outperforms the brittle Al_2_O_3_ layer as an interfacial bridge between Cu and PET (Figure 1), enabling 5B-level interfacial adhesion via strong chemical anchoring and inducing the growth of dense, (111)-textured Cu films. The optimized Cu/NiCr thin films exhibit exceptional electromechanical durability (R/R_0_ < 5% after 1000 bending cycles) and remarkable oxidation resistance under damp-heat aging conditions (85 °C/85% RH). Leveraging maskless laser lithography, we further fabricate Cu meshes with high optical transmittance (87%) and low sheet resistance (10.8 Ω/sq) on the optimized Cu/NiCr/PET structure, and validate their practical viability in flexible transparent heaters with rapid thermal response and long-term operational stability (>20 h of continuous operation).

## 2. Experimental Details

### 2.1. Fabrication of Cu Thin Films and Mesh Electrodes

Commercial polyethylene terephthalate (PET) films (~50 µm thick) are used as flexible substrates. Three distinct types of Cu thin films are prepared to systematically investigate the effect of interfacial layers on film performance: (1) Cu thin films without an interfacial layer; (2) Cu thin films with a NiCr interfacial layer; (3) Cu thin films with an Al_2_O_3_ interfacial layer. Prior to deposition, the samples are loaded into a magnetron sputtering chamber (base pressure ≈ 8 × 10^−4^ Pa) and pretreated with in situ argon (Ar) plasma (1 Pa, 2 min) to enhance surface energy. The entire deposition sequence—including plasma activation, ultrathin interfacial layer growth (~6 nm), and the subsequent 210 nm Cu layer deposition—was sequentially executed in the same vacuum chamber. For interfacial layer deposition, the NiCr layer (~6 nm) is deposited via pulsed-DC sputtering (50 W, 0.33 Pa Ar), while the Al_2_O_3_ interfacial layer (~6 nm) is prepared via reactive sputtering at 70 W under a working pressure of 1 Pa, with an Ar:O_2_ gas flow ratio of 45:6 and a deposition time of 72 s. A Cu functional layer with a thickness of approximately 210 nm is then deposited on all samples using a polycrystalline Cu target via DC sputtering (200 W, 0.46 Pa Ar).

Following the optimization of the thin films, high-precision Cu mesh electrodes are fabricated using the NiCr and Al_2_O_3_ interfacial layer via a photolithography-assisted lift-off process. A positive photoresist (Bynano, AZ-5214, Xi’an, China) is spin-coated onto the cleaned PET substrates and soft-baked at 95 °C for 2 min, yielding a uniform resist thickness of ~1.4 µm. Unlike traditional mask alignment, the mesh patterns are defined using a desktop maskless direct-writing lithography system (Durham Magneto Optics, MicroWriter ML3, Cambridge, UK) based on Digital Micromirror Device (DMD) projection. The system operates with a 405 nm laser source (nominal optical power of 1.5 W) and is controlled by a edited electronic mask program. Exposure is performed in the 5 µm mode (corresponding to an effective pixel/spot size of ~5 µm) with an exposure dose correction setting of 0.8. Notably, this DMD-based projection strategy exhibits high throughput and scalability; a representative 5 cm × 5 cm area (25 cm^2^) is patterned within 30 min, corresponding to an effective patterning rate of ~83.3 mm^2^/min. The achieved feature size exhibits a precise line width of 10 µm and an average period of ~500 µm. After exposure, the samples are developed in ZA-238 positive developer for 45 s, rinsed with deionized water, and dried with N_2_. Subsequently, the optimal film stack (Ar plasma activation, NiCr and Al_2_O_3_ interfacial layer, and Cu layer) is sequentially deposited under the aforementioned conditions. Finally, the lift-off process is performed by immersing the samples in acetone for 2 h followed by ultrasonication for 10 min to remove the photoresist and excess metal. The samples are stored at room temperature prior to optoelectronic and mechanical testing.

Two distinct aperiodic mesh geometries are designed and fabricated to optimize the trade-off between optoelectronic efficiency and mechanical flexibility: an aperiodic Linear mesh (Linear) composed of straight ligaments, and a stress-relieving aperiodic Sinusoidal mesh (Sinusoidal) featuring curved interconnected pathways. Both geometries are fabricated using the optimized NiCr layer process.

### 2.2. Characterization

The film thickness is determined via spectroscopic ellipsometry (J.A. Woollam, M2000DI, Lincoln, NE, USA). For structural and morphological characterization, the crystal structure is analyzed using X-ray diffraction (XRD, Bruker AXS D8 Advance, Bruker, Billerica, MA, USA) with Cu Kα radiation, while the surface morphologies and the quality of the mesh patterns are examined using cold field-emission scanning electron microscopy (FE-SEM, Hitachi S-4800, Hitachi, Tokyo, Japan). Detailed interfacial microstructure and elemental distribution analyses are performed using a transmission electron microscope (TEM, FEI Tecnai F20, Hillsboro, OR, USA) equipped with an energy-dispersive X-ray spectroscopy (EDS) system at an accelerating voltage of 200 kV. The surface roughness of the different interfacial layers is quantified by atomic force microscopy (AFM, Veeco Dimension 3100 V, Plainview, NY, USA). Interfacial adhesion was quantitatively evaluated through cross-cut tape tests. A multi-blade cutter was used to create a grid of 1 mm spacing on the film surface. After removing debris, a standard pressure-sensitive tape was applied and then peeled off steadily at an angle of ~180°. The adhesion strength was classified from 0B to 5B based on the area of delamination, following the ASTM D3359-23 Method B grading system [20].

Regarding optoelectronic properties, the sheet resistance is measured using a four-point probe system (NAPSON, Cresbox, Tokyo, Japan), and transmittance spectra are acquired using a UV-vis-NIR spectrophotometer (PerkinElmer Lambda950, Waltham, MA, USA) equipped with an integrating sphere. Hall effect measurements are conducted to analyze carrier transport properties (mobility and concentration). The mechanical durability under repeated bending is evaluated using a flexible testing system (NAPSON, FlexTest mini-F-C, Changsha, China) with a bending radius of 2 mm and a bending angle of 60°. Damp-heat aging tests are performed at 85 °C and 85% relative humidity (RH) to evaluate oxidation resistance and environmental tolerance, with electrical performance and surface morphology monitored every 20 min over a duration of 100 min.

For the practical application evaluation in flexible transparent heaters (FTHs), copper foil electrodes are attached to opposite edges of the Linear and Sinusoidal mesh films to ensure uniform current distribution. The time-dependent temperature profiles under a 5 V step voltage are recorded, and multi-voltage step heating experiments (3 V to 7 V) are conducted to assess thermal output controllability. Long-term operational reliability is evaluated under a constant bias of 5 V for over 20 h.

## 3. Results and Discussion

### 3.1. Influence of Interfacial Layers on Interfacial Stability and Microstructural Evolution

**Figure 2:**
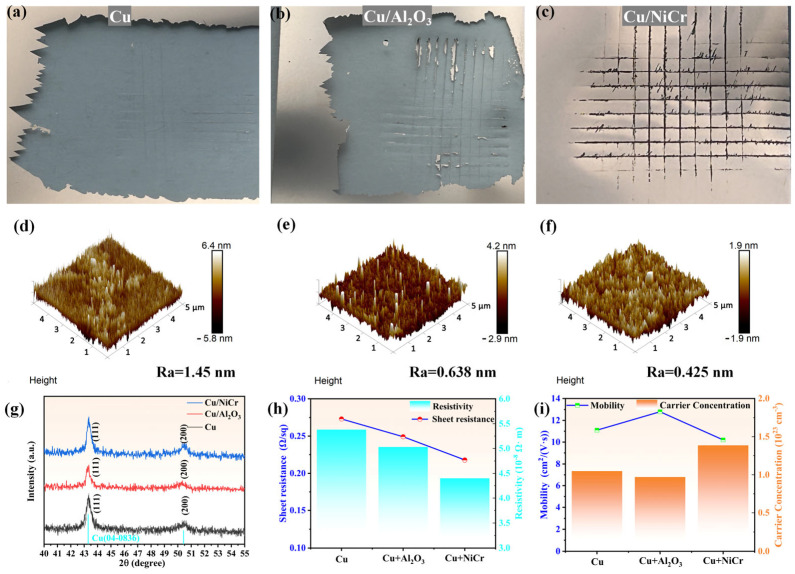
Interfacial adhesion and microstructural characterization of Cu thin films with different interfacial layers. (**a**–**c**) Optical micrographs of cross-cut tape test results for (**a**) pristine Cu (seedless), (**b**) Cu/Al_2_O_3_, and (**c**) Cu/NiCr thin films. (**d**) Comparison of sheet resistance and electrical resistivity for the three thin film systems. (**e**) Carrier transport properties (mobility and concentration) derived from Hall effect measurements. (**f**) XRD patterns. (**g**–**i**) 3D-AFM topographic images and corresponding arithmetic average roughness (Ra) values for (**g**) pristine Cu, (**h**) Cu/Al_2_O_3_, and (**i**) Cu/NiCr.

The robust integration of metallic thin films onto flexible polymer substrates is a prerequisite for reliable flexible optoelectronics, yet the chemical inertness of PET often leads to weak interfacial adhesion [3,21]. Standard cross-cut tape tests are conducted to quantitatively evaluate the efficacy of different interfacial layers, as shown in Figure 2a–c. The Cu without layer deposited directly on the PET substrate suffers from severe delamination, with the majority of the grid area peeling off. This failure is primarily attributed to the low surface energy of the polymer and the lack of reactive sites, resulting in weak physical adsorption. The introduction of an Al_2_O_3_ layer offers a marginal improvement, but extensive detachment persists along the cutting edges, indicating that the oxide-polymer interaction remains insufficient to withstand mechanical stress [22,23]. In contrast, the incorporation of the metallic NiCr interfacial layer fundamentally reinforces the interfacial adhesion; the Cu/NiCr thin film maintains a pristine grid structure with sharp, intact edges, successfully achieving the highest 5B classification (0% delamination area) under the standard criteria. This superior bonding is ascribed to the synergistic effect of Ar plasma activation and the metallic NiCr layer, which facilitates the formation of strong interfacial chemical bonds (e.g., Ni-O/Cr-O), thereby effectively anchoring the Cu layer to the substrate [24]. Atomic force microscopy (AFM) analysis further reveals that the interfacial layers significantly suppress surface roughness, as shown in Figure 2d–f. The Cu without layer (Figure 2d) exhibits a coarse morphology with a relatively high arithmetic average roughness (R_a_) of 1.45 nm, indicative of a Volmer–Weber island growth mode [25,26]. The Cu/Al_2_O_3_ sample (Figure 2e) shows a reduced roughness of 0.64 nm, while the Cu/NiCr sample (Figure 2f) displays the densest and smoothest surface architecture, with the R_a_ value decreasing to a minimum of 0.43 nm. This ultra-smooth surface suggests that the NiCr layer promotes uniform nucleation and planar growth [27,28]. Correspondingly, X-ray diffraction (XRD) patterns demonstrate that all thin films exhibit characteristic face-centered cubic (fcc) Cu (111) and (200) peaks, as shown in Figure 2g. However, the Cu/NiCr thin film exhibits the sharpest and most intense (111) diffraction peak [29,30]. Calculations based on the Scherrer equation indicate that the average crystallite size increases from approximately 16.6 nm for the Cu without layer to 20.8 nm for the Cu/Al_2_O_3_ thin film and 21.1 nm for the Cu/NiCr thin film. This coarsening of grains confirms that the NiCr layer not only enhances adhesion but also promotes the texturing of Cu grains along the (111) orientation, resulting in a highly crystalline microstructure. The electrical properties of the Cu thin films are intrinsically governed by these microstructural characteristics [25,30]. The Cu/NiCr thin film exhibits superior electrical performance (Figure 2h), achieving the lowest sheet resistance of 0.218 Ω/sq and a resistivity of 4.40 µΩ·cm. In comparison, the Cu/Al_2_O_3_ and Cu without layer display higher resistivity values of 5.03 µΩ·cm and 5.38 µΩ·cm, respectively. Hall effect measurements reveal distinct differences in mobility and carrier concentration (Figure 2i). The Cu without layer displays a carrier mobility of 11.1 cm^2^/V·s and a carrier concentration of 1.05 × 10^23^ cm^−3^. Upon introducing the Al_2_O_3_ layer, the mobility increases to 12.8 cm^2^/V·s, attributed to the suppression of surface roughness scattering [31]. Conversely, the Cu/NiCr thin film exhibits a slightly reduced mobility of 10.2 cm^2^/V·s, despite possessing the smoothest surface. This reduction implies that while surface scattering is minimized, the diffusion of Ni and Cr atoms into the Cu lattice likely introduces point defects, acting as impurity scattering centers that impede electron transport [32,33]. Nevertheless, the Cu/NiCr thin film demonstrates a substantial increment in carrier concentration, reaching a maximum of 1.42 × 10^23^ cm^−3^. This high carrier density effectively offsets the mobility loss, thereby serving as the dominant factor responsible for the minimized resistivity.

**Figure 3:**
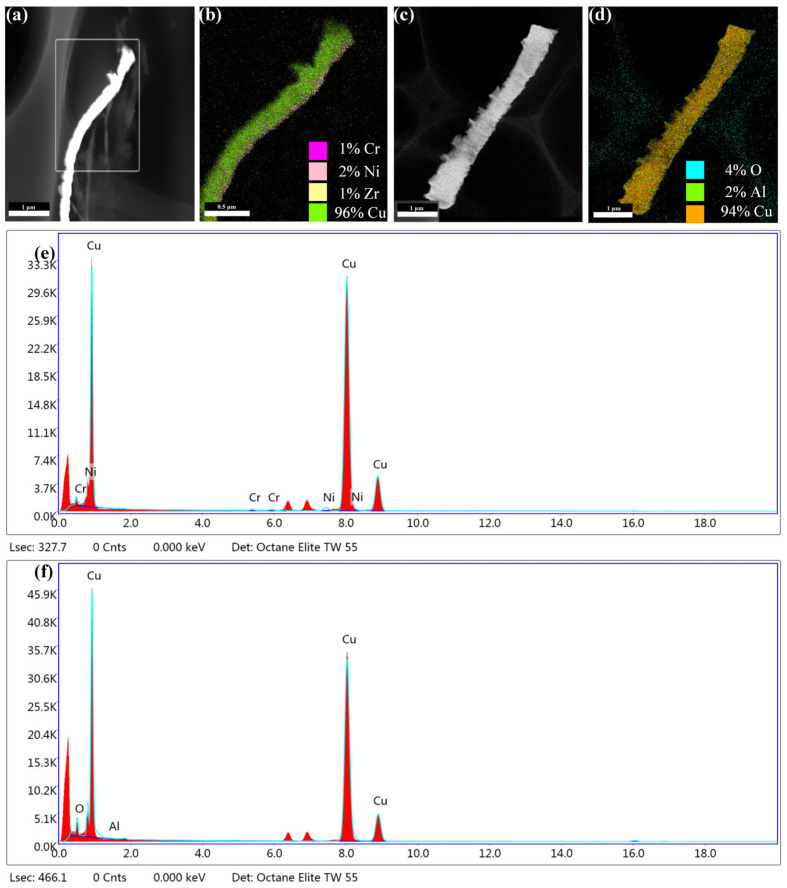
Elemental composition and interfacial analysis of Cu thin films with Al_2_O_3_ and NiCr interfacial layers. (**a**) STEM image of the Cu/NiCr sample. (**b**) Corresponding EDS elemental map showing Cu together with interfacial enrichment of Ni and Cr. (**c**) STEM image of the Cu/Al_2_O_3_ sample. (**d**) Corresponding EDS elemental map showing Cu with co-detected Al and O signals. (**e**,**f**) EDS spectra acquired from the mapped regions for (**e**) Cu/NiCr and (**f**) Cu/Al_2_O_3_ samples, respectively.

Energy-dispersive X-ray spectroscopy (EDS) elemental mapping and spectral analysis were performed to verify the elemental composition and confirm the incorporation of the interfacial layers (Figure 3). For the Cu/Al_2_O_3_ thin film, Al and O signals are detected together with the Cu matrix in the EDS map (Figure 3d) and are further supported by the corresponding EDS spectrum (Figure 3f), confirming the presence of Al_2_O_3_-derived species. Notably, the Al/O signals are not confined to a sharp, well-defined interfacial band, which may be attributed to the ultrathin/discontinuous nature of the oxide layer and/or growth-induced mixing during Cu deposition.

For the Cu/NiCr sample, although the Ni and Cr signals in the STEM-EDS elemental maps (Figure 3b) appear relatively faint due to the ultrathin nature of the interfacial layer (~6 nm), their existence is unequivocally confirmed by the corresponding EDS spectrum (Figure 3e). Quantitative analysis reveals trace concentrations of 1.42 wt% (1.53 at%) for Ni and 1.01 wt% (1.23 at%) for Cr (Table S1), which sufficiently verifies the successful construction of the targeted Cu/NiCr interfacial architecture. Moreover, the presence and interfacial enrichment of Ni and Cr support the proposed mechanism (Figure 1), wherein solute atoms reinforce the interface and modulate the nucleation kinetics of the overlying Cu film [34].

The superior adhesion of the Cu/NiCr interface is attributed to the synergistic interaction of both nickel (Ni) and chromium (Cr) elements with the substrate. Upon deposition on the plasma-activated PET surface, the high-energy metallic atoms (Ni and Cr) collectively react with the abundant oxygen-containing functional groups (e.g., C=O, –OH), establishing a robust chemical bonding network (metal–O–C). Specifically, Cr serves as a reactive anchor by forming strong covalent bonds due to its high oxygen affinity, while Ni, as the chemically stable matrix, promotes the formation of a dense, continuous interfacial layer that physically interlocks with the polymer surface [8]. This cooperative mechanism effectively prevents the localized delamination often observed in single-component interfaces, ensuring 5B-level adhesion strength. Furthermore, unlike the abrupt boundary formed with ceramic interfacial layers (e.g., Al_2_O_3_), the Cu/NiCr interface facilitates the formation of a metallic solid solution. Since Copper (Cu) and Nickel (Ni) share the same face-centered cubic (FCC) crystal structure and similar atomic radii, they exhibit complete solid solubility. This allows for the interdiffusion of Ni and Cu atoms, creating a graded transition zone rather than a sharp interface [35]. As evidenced by the EDS mapping (Figure 3b), this interdiffusion layer effectively bridges the lattice mismatch and enhances interfacial toughness, thereby suppressing void formation and crack initiation under mechanical stress.

### 3.2. Electromechanical Reliability and Environmental Stability

**Figure 4:**
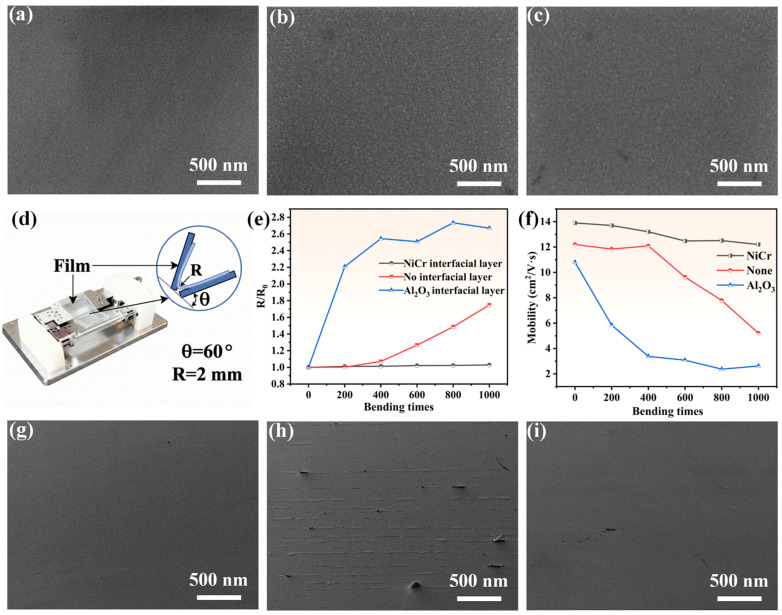
Electromechanical reliability of Cu thin films under dynamic bending deformation. (**a**–**c**) FE-SEM images of as-deposited (**a**) pristine Cu, (**b**) Cu/Al_2_O_3_, and (**c**) Cu/NiCr thin films. (**d**) Schematic of the dynamic bending test setup: bending radius (R) = 2 mm, bending angle (θ) = 60°, and cyclic deformation up to 1000 cycles. (**e**) Normalized resistance change (R/R_0_) as a function of bending cycles. (**f**) Evolution of Hall mobility during bending tests. (**g**–**i**) Post-bending FE-SEM images after 1000 cycles: (**g**) pristine Cu with visible cracks and delamination, (**h**) Cu/Al_2_O_3_ with dense transverse microcracks, and (**i**) Cu/NiCr retaining a continuous, crack-free surface.

The surface morphology and electromechanical stability of the films under dynamic deformation are systematically investigated. Scanning electron microscopy (SEM) images of the as-deposited thin films show that both the Cu thin film without a interfacial layer and the Cu/Al_2_O_3_ thin film exhibit a granular morphology characterized by discernible voids and loose grain boundaries (Figure 4a–c). In contrast, the Cu/NiCr thin film displays a highly compact, pinhole-free, and continuous surface architecture, confirming that the metallic NiCr layer promotes high-quality, dense thin film growth [8,27]. Dynamic bending tests with a bending radius (R) of 2 mm and a bending angle of 60° reveal distinct degradation behaviors among the three samples, as shown in Figure 4d–f. The Cu/Al_2_O_3_ thin film undergoes rapid electromechanical degradation, with the normalized resistance (R/R_0_) surging to ≈2.7 after only 400 cycles. This abrupt failure is attributed to the inherent brittleness of the ceramic oxide layer, which is prone to fracture under tensile stress, subsequently severing the conductive Cu network [36]. The Cu thin film without a interfacial layer shows a gradual increase in resistance, reaching R/R_0_ ≈ 1.8 after 1000 cycles, primarily due to the accumulation of fatigue cracks and localized delamination caused by weak interfacial adhesion. Remarkably, the Cu/NiCr thin film demonstrates exceptional durability, with the resistance remaining negligible (R/R_0_ ≈ 1.0) throughout the 1000 bending cycles. The evolution of Hall mobility during bending further elucidates the microscopic degradation mechanism. For the Cu/NiCr thin film, the mobility is maintained at a stable level, indicating that the electron transport channels remain intact without significant scattering induced by structural defects. Conversely, the mobility of the Cu/Al_2_O_3_ thin film exhibits a precipitous decay, confirming that the generation of high-density cracks acts as a severe scattering source for charge carriers. Post-bending SEM characterization corroborates these findings (Figure 4g–i): the bent Cu/Al_2_O_3_ surface is characterized by dense, transverse microcracks, the Cu thin film without a interfacial layer exhibits visible crack propagation and signs of localized detachment, while the Cu/NiCr thin film retains a smooth, continuous, and crack-free morphology even after 1000 cycles. This superior bending fatigue resistance confirms that the ductile NiCr alloy layer not only strengthens interfacial adhesion but also effectively dissipates mechanical stress, thereby suppressing crack initiation and propagation [32].

**Figure 5:**
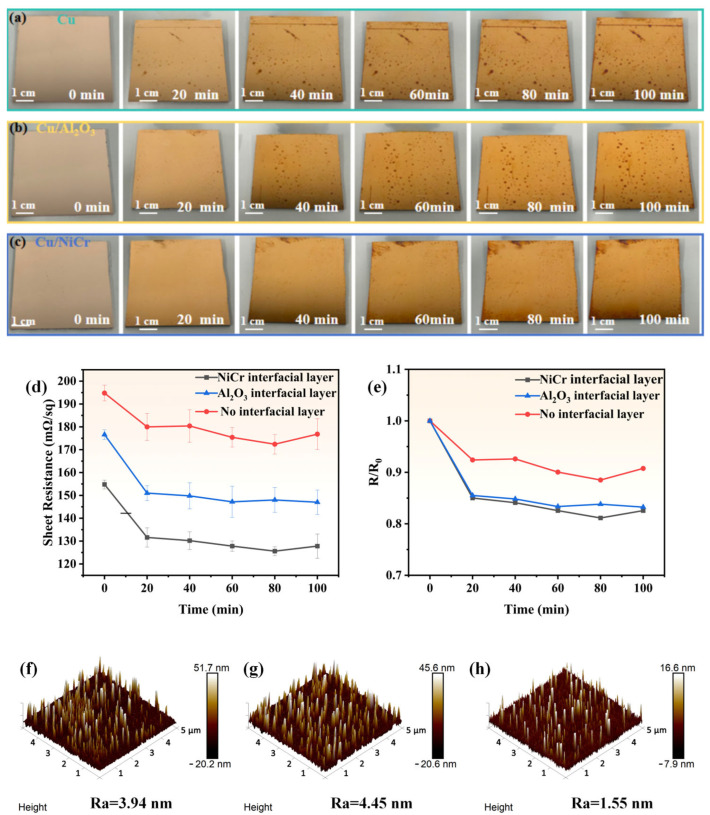
Environmental stability of Cu thin films under damp-heat aging conditions (85 °C/85% RH). (**a**–**c**) Macroscopic optical images of (**a**) pristine Cu, (**b**) Cu/Al_2_O_3_, and (**c**) Cu/NiCr thin films recorded at aging intervals from 0 to 100 min. (**d**) Sheet resistance and (**e**) normalized resistance (R/R_0_) variations as a function of aging time, with the Cu/NiCr thin film exhibiting the highest stability. (**f**–**h**) Post-aging 3D AFM images and roughness (R_a_) analysis: (**f**) pristine Cu (R_a_ = 3.94 nm), (**g**) Cu/Al_2_O_3_ (R_a_ = 4.45 nm), and (**h**) Cu/NiCr (R_a_ = 1.55 nm).

Damp-heat aging tests at 85 °C and 85% relative humidity (RH) are conducted to evaluate the long-term operational reliability of the films under harsh environmental conditions, as shown in Figure 5. The Cu thin film without a interfacial layer and the Cu/Al_2_O_3_ thin film exhibit pronounced oxidative degradation, with visible oxidation spots and localized corrosion pits emerging rapidly within the first 40 min and expanding significantly as the aging time extends. This severe surface discoloration suggests that moisture and oxygen molecules easily penetrate the loosely packed grain boundaries, facilitating oxidative corrosion [29,37,38]. In contrast, the Cu/NiCr thin film demonstrates significantly enhanced resistance to environmental corrosion, with only minor, localized oxidation spots observed after prolonged exposure. The quantitative evolution of electrical properties during aging shows that all samples exhibit a decreasing trend in sheet resistance (Rs) and normalized resistance (R/R_0_) during the initial stage, primarily attributed to the thermal annealing effect at 85 °C, which promotes grain growth and defect annihilation [39]. However, the magnitude of this change varies significantly: the Cu thin film without a interfacial layer and Cu/Al_2_O_3_ thin film show a sharp resistance drop (R/R_0_ decreases to ≈0.8), indicative of the annealing of a high density of pre-existing defects and structural instability under thermal stress. Conversely, the Cu/NiCr thin film exhibits the highest stability, with the resistance showing only a marginal fluctuation (R/R_0_ stabilizes around 0.9). Post-aging AFM characterization further corroborates the protective mechanism of the NiCr layer: the surfaces of the aged Cu thin film without a interfacial layer and Cu/Al_2_O_3_ thin film appear rough and uneven, with R_a_ values increasing to 3.94 nm and 4.45 nm, respectively, while the Cu/NiCr thin film retains a remarkably smooth and dense surface topology with the lowest R_a_ of 1.55 nm. This result confirms that the dense packing of Cu (111) grains induced by the NiCr layer acts as an effective diffusion barrier against water vapor and oxygen, thereby significantly enhancing the environmental lifespan of the electrode [38].

### 3.3. Geometric Engineering for Electromechanical Optimization

Conventional periodic metal meshes often suffer from coherent optical interference, leading to distinct diffraction artifacts and Moiré patterns when overlaid on pixel arrays [40]. Aperiodic or random mesh designs can effectively suppress these coherent diffraction effects, minimizing Moiré patterns to achieve superior optical homogeneity and visual invisibility [41,42]. Quantitative comparisons from the literature are compiled in the Supporting Information (Table S2). These reports indicate that introducing aperiodicity reduces the non-uniformity of high-order diffraction (Cv) and weakens concentrated high-order stray-light components, which in turn alleviates diffraction-related artifacts and Moiré visibility. The reported sheet resistance remains comparable to, or better than, that of periodic meshes. Further numerical details and references are provided in Table S2.

**Figure 6:**
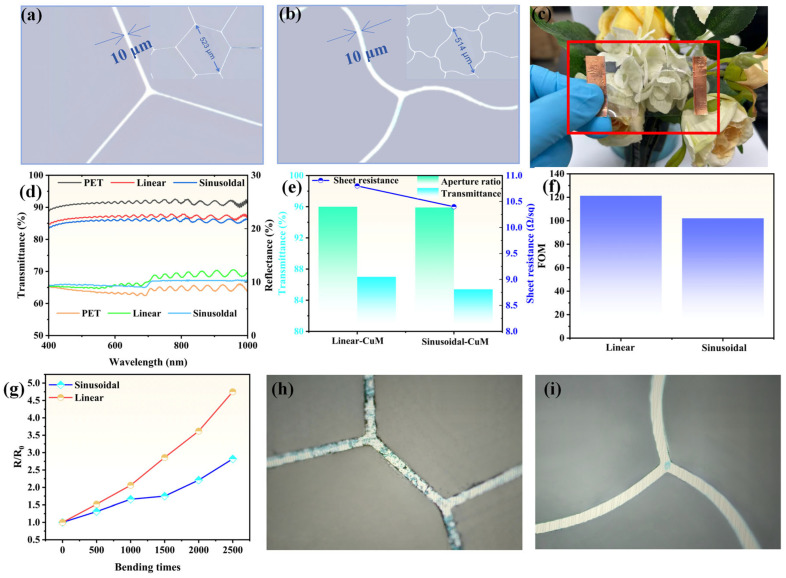
Geometric engineering of aperiodic Cu meshes for optimized electromechanical performance. (**a**,**b**) Metallographic micrographs of (**a**) Linear and (**b**) Sinusoidal aperiodic mesh patterns. (**c**) Photograph of the Sinusoidal mesh electrode sample. (**d**) UV-vis-NIR transmittance spectra of bare PET, Linear, and Sinusoidal thin films in the 400–1000 nm wavelength range. (**e**) Comparison of sheet resistance and transmittance at 550 nm for the two mesh geometries. (**f**) Calculated Figure of Merit (FoM) highlighting the superior optoelectronic balance of the Sinusoidal mesh. (**g**) Normalized resistance change (R/R_0_) as a function of bending cycles (R = 2 mm). (**h**,**i**) Post-bending metallographic micrographs after 2500 cycles: (**h**) Linear mesh with severe transverse fractures at hexagonal vertices, and (**i**) Sinusoidal mesh retaining structural integrity without observable cracks.

Motivated by this optical advantage, an aperiodic tessellation strategy is adopted for the electrode design, with two distinct aperiodic mesh geometries fabricated: an aperiodic Linear mesh (Linear) and a stress-relieving aperiodic Sinusoidal mesh (Sinusoidal) [43]. Metallographic micrographs (Figure 6a,b) show that both the Linear and Sinusoidal samples exhibit highly uniform network structures with well-defined edges, comparable to commercial metal mesh films. A macroscopic photograph (Figure 6c) visually demonstrates the excellent optical clarity of the mesh electrodes, allowing for distinct visibility of the background features. The theoretical optical aperture ratio (AR) is calculated based on the geometric lattice definition, and the Figure of Merit (FoM) is calculated using the ratio of DC conductivity to optical conductivity [44]. In terms of static optoelectronic properties, the Linear mesh exhibits a sheet resistance (Rs) of 10.8 Ω/sq, an aperture ratio of 96%, and a high transmittance of 87.0% at 550 nm, as shown in Figure 6d–f. In comparison, as summarized in Table 1, the Sinusoidal mesh demonstrates a marginally lower sheet resistance of 10.4 Ω/sq and an aperture ratio of 95.9%, but its transmittance is lower at 85.7%. Consequently, despite the slightly higher resistance, the Linear mesh yields a superior FoM of 121.3, compared to 102.1 for the Sinusoidal mesh. This quantitative analysis indicates that the Linear design, with its higher aperture ratio and transmittance, offers a higher static optoelectronic performance. Bending fatigue tests reveal a stark contrast in stability between the two geometries, as shown in Figure 6g–i. The Linear mesh undergoes rapid electromechanical degradation, with the normalized resistance (R/R_0_) surging to ≈4.7 after 2500 bending cycles. Post-bending microscopic analysis shows severe transverse fractures concentrated at the sharp hexagonal vertices, severing the electrical network. In contrast, the Sinusoidal mesh demonstrates superior durability, with the resistance increase significantly suppressed (R/R_0_ ≈ 2.8) under identical conditions. The Sinusoidal structure remains continuous without observable cracking as the Sinusoidal curvature effectively acts as a mechanical spring, delocalizing tensile stress along the arcs rather than concentrating it at the junctions [45,46]. Consequently, the systematic comparison reveals a crucial practical trade-off for device design. The aperiodic Linear mesh maximizes the optical aperture, offering superior static optoelectronic efficiency that is ideal for stationary applications. Conversely, the Sinusoidal mesh functions as a two-dimensional mechanical spring, effectively delocalizing stress to provide a critical enhancement in electromechanical reliability. This geometric compliance renders the Sinusoidal design the unequivocally preferred candidate for highly flexible and wearable electronics, justifying the marginal compromise in static optical clarity.

### 3.4. Practical Application in Flexible Transparent Heaters

**Figure 7:**
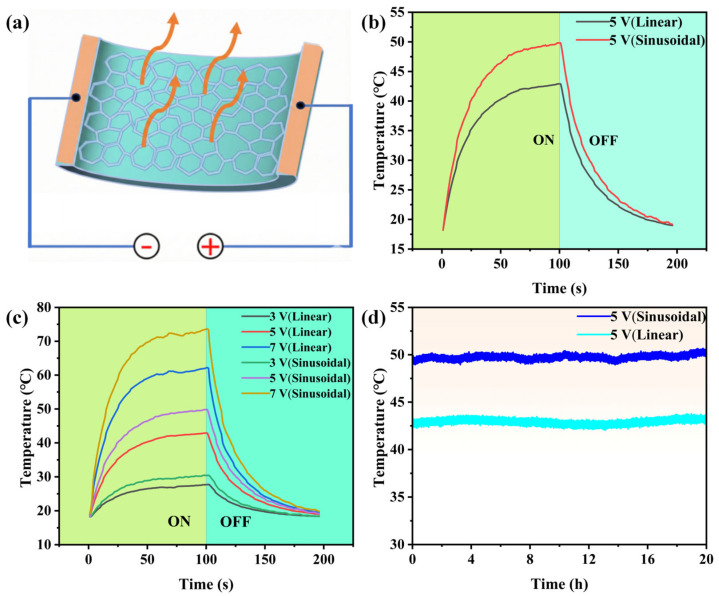
Performance of flexible transparent heaters (FTHs) based on Linear and Sinusoidal Cu mesh electrodes. (**a**) Schematic diagram of the FTH device structure: Cu mesh electrode on PET, with copper foil current collectors attached to opposite edges. (**b**) Time-dependent temperature profiles under a constant applied voltage of 5 V. (**c**) Temperature response curves under stepwise voltage increases (3 V to 7 V). (**d**) Long-term operational stability test at 5 V for 20 h.

To validate the practical viability of the fabricated electrodes, flexible transparent heaters (FTHs) are assembled using Linear and Sinusoidal thin films (Figure 7) [47]. The time-dependent temperature profiles under a 5 V step voltage reveal a rapid thermal response: the surface temperature rises from room temperature (∼20 °C) to thermal equilibrium within approximately 40 s and swiftly returns to the initial state upon power-off. The Linear heater achieves a higher saturation temperature of 49.8 °C, whereas the Sinusoidal heater stabilizes at 42.9 °C. This discrepancy is governed by the Joule heating mechanism (P = V^2^/R), where the lower sheet resistance of the Linear mesh generates a higher power density compared to the Sinusoidal counterpart. Multi-voltage step heating experiments demonstrate precise controllability of the thermal output. As the applied voltage is incrementally stepped from 3 V to 7 V, the saturation temperature of the Linear heater increases monotonically from 30.4 °C to a maximum of 73.6 °C. Similarly, the Sinusoidal heater exhibits stable temperature plateaus ranging from 27.7 °C to 62.1 °C. The observed quadratic dependence of temperature on input voltage enables accurate, wide-range thermal tuning suitable for applications such as defogging systems and wearable thermotherapy [43,48]. Long-term operational reliability evaluated under a constant bias of 5 V for over 20 h shows that both devices exhibit exceptional stability, characterized by negligible resistance drift and constant thermal output throughout the testing duration. The maintenance of stable heating profiles (Linear at ∼50 °C and Sinusoidal at ∼43 °C) confirms robust resistance against thermal degradation. This superior electro-thermal stability is attributed to the dense NiCr layer, which acts as a robust diffusion barrier, effectively mitigating oxidation kinetics and preventing the inter-diffusion of species at elevated temperatures [49]. These results confirm that both mesh designs offer viable technical solutions for high-performance heaters, with the Linear design maximizing thermal efficiency and the Sinusoidal design offering a superior balance of mechanical compliance and optoelectronic performance.

## 4. Conclusions

This work presents a robust alloyed interface engineering strategy to address the critical interfacial adhesion weakness and inadequate mechanical durability of Cu-based flexible transparent conductive electrodes (TCEs) on PET substrates. Systematic comparative studies of ceramic (Al_2_O_3_) and metallic (NiCr) interfacial layers reveal that the NiCr layer forms a distinct alloyed interface with Cu via interdiffusion, which fundamentally reinforces the Cu/PET interface. The optimized Cu/NiCr thin films achieve 5B-level adhesion, a low sheet resistance of 0.218 Ω/sq, exceptional electromechanical durability (R/R_0_ < 5% after 1000 bending cycles), and remarkable environmental stability under damp-heat conditions. By leveraging maskless photolithography, Cu is precisely patterned into micrometer-scale aperiodic meshes. Among the designed geometries, the Linear mesh exhibits superior optoelectronic performance, achieving ~10.8 Ω/sq sheet resistance, >87% optical transmittance, and a high FoM of 121.3, while the Sinusoidal mesh offers a superior mechanical flexibility. Practical validation in flexible transparent heaters (FTHs) confirms rapid thermal response, precise temperature controllability, and long-term operational stability (>20 h), underscoring the electrodes’ potential for next-generation flexible optoelectronics. Microstructural analysis clarifies the synergistic toughening mechanism, showing that the gradient alloyed zone formed at the Cu-NiCr interface effectively mitigates interfacial stress concentrations and suppresses crack propagation. This work provides a viable pathway for developing high-performance, durable flexible TCEs, offering valuable insights for the design and optimization of next-generation flexible electronic devices.

## Figures and Tables

**Table 1:** Optoelectronic properties and FoM of Cu mesh electrodes with different geometric parameters.

Sample	Rs (Ω/sq)	T (550 nm) (%)	Aperture Ratio	FoM
Linear	10.8	87.0	96%	121.3
Sinusoidal	10.4	85.7	95.9%	102.1

## Data Availability

The original contributions presented in this study are included in the article/Supplementary Materials. Further inquiries can be directed to the corresponding authors.

## Supplementary Materials

The following supporting information can be downloaded at: https://www.mdpi.com/article/10.3390/ma19122608/s1, Table S1. Quantitative elemental composition of the Cu thin films with Al_2_O_3_ and NiCr seed layers derived from EDS spectral analysis. Table S2. Quantitative comparison between periodic (regular) and aperiodic (random) metal meshes. References [50,51,52,53] are cited in the Supplementary Materials.
