# Supplementary material for: Optimized Interfacial Layers for High-Adhesion and Damp-Heat-Resistant Cu Meshes with Aperiodic Geometries on PET Substrates
Source: Materials (Basel). 2026 Jun 17;19(12):2608. doi: 10.3390/ma19122608 (PMC13303418; doi:10.3390/ma19122608)
Supplement: Supplementary file 1 [file materials-19-02608-s001.zip › materials-4178576-supplementary.pdf]

Supporting Information

Table S1. Quantitative elemental composition of the Cu thin films with Al_2O_3 and NiCr seed layers derived from EDS spectral analysis.

Sample	Element	Weight (%)	Atomic (%)
Cu/Al_2O_3	Cu	94.28	97.73
	Al	2.21	1.36
	O	3.51	1.07
Cu/NiCr	Cu	97.57	97.24
	Ni	1.42	1.53
	Cr	1.01	1.23

Table S1 presents a comparative analysis of EDS spectra for Cu thin films using Al_2O_3 and NiCr seed layers, respectively. Energy-dispersive spectroscopy (EDS) elemental mapping and quantitative analysis confirm the elemental composition and verify the successful integration of the interfacial layers. For the Cu/Al_2O_3 thin film, elemental mapping reveals a homogeneous distribution of aluminum (Al) and oxygen (O) within the copper (Cu) matrix, with an Al content of 2.21 wt% (1.36 at%), demonstrating the effective deposition of the oxide layer. For the Cu/NiCr sample, distinct signals corresponding to nickel (Ni) and chromium (Cr) are clearly identified, with respective concentrations of 1.42 wt% (1.53 at%) and 1.01 wt% (1.23 at%).

Table S2. Quantitative comparison between periodic (regular) and aperiodic (random) metal meshes.

Metric	Regular Mesh (Periodic)	Random / Aperiodic Mesh	Improvement	Ref
Diffraction non-uniformity Cv	1588.60%	397.95%	↓ ~75%	[1]
High-order stray-light energy	High	Low	Reduced	[2]
Moiré visibility	Strong	Weak	Eliminated	[3]
Sheet resistance Rs	<10 Ω/Sq	<1 Ω/Sq	Comparable or better	[4]

Table S2 summarizes the performance difference between periodic and aperiodic metal meshes. Aperiodic meshes markedly reduce high-order diffraction non-uniformity (Cv) and suppress concentrated high-order stray-light components, thereby mitigating diffraction-related artifacts. They also alleviate moiré visibility while maintaining sheet resistance comparable to or better than periodic designs.

References

[1] Zhong H, Han Y, Lin J, Jin P. Pattern randomization: an efficient way to design high-performance metallic meshes with uniform stray light for EMI shielding. Opt Express 2020;28:7008.

[2] Lu Z, Wang H, Tan J, Ma L, Lin S. Achieving an ultra-uniform diffraction pattern of stray light with metallic meshes by using ring and sub-ring arrays. Opt Lett 2016;41:1941.

[3] Shin D-K, Park J. Suppression of Moiré phenomenon induced by metal grids for touch screen panels. J Display Technol 2016;12:632–8.

[4] Jiang Z, Huang W, Chen L, Liu Y. Ultrathin, lightweight, and freestanding metallic mesh for transparent electromagnetic interference shielding. Opt Express 2019;27:24194.
